# Maternal choline supplementation mitigates premature foetal weight gain induced by an obesogenic diet, potentially linked to increased amniotic fluid leptin levels in rats

**DOI:** 10.1038/s41598-024-62229-2

**Published:** 2024-05-18

**Authors:** Zhi Xin Yau-Qiu, Sebastià Galmés, Pedro Castillo, Catalina Picó, Andreu Palou, Ana María Rodríguez

**Affiliations:** 1 Laboratory of Molecular Biology, Nutrition and Biotechnology (Group of Nutrigenomics, Biomarkers and Risk Evaluation-NuBE), University of the Balearic Islands (UIB), Cra. Valldemossa Km 7.5, 07122 Palma, Balearic Islands Spain; 2 Health Research Institute of the Balearic Islands (IdISBa), 07120 Palma, Spain; 3 CIBER of Physiopathology of Obesity and Nutrition (CIBEROBN), Instituto de Salud Carlos III, 28029 Madrid, Spain

**Keywords:** Leptin, Perinatal nutrition, Foetal development, Choline, Sex-dependent metabolic programming, RNA, Nutrition

## Abstract

Placental leptin may impact foetal development. Maternal overnutrition has been linked to increased plasma leptin levels and adverse effects on offspring, whereas choline, an essential nutrient for foetal development, has shown promise in mitigating some negative impacts of maternal obesity. Here, we investigate whether a maternal obesogenic diet alters foetal growth and leptin levels in the foetal stomach, amniotic fluid (AF), and placenta in late gestation and explore the potential modulating effects of maternal choline supplementation. Female rats were fed a control (CD) or a western diet (WD) four weeks before mating and during gestation, half of them supplemented with choline (pregnancy days 11–17). Leptin levels (in foetal stomach, AF, and placenta) and leptin gene expression (in placenta) were assessed on gestation days 20 and 21. At day 20, maternal WD feeding resulted in greater leptin levels in foetal stomach, placenta, and AF. The increased AF leptin levels were associated with a premature increase in foetal weight in both sexes. Maternal choline supplementation partially prevented these alterations, but effects differed in CD dams, causing increased AF leptin levels and greater weight in male foetuses at day 20. Maternal choline supplementation effectively mitigates premature foetal overgrowth induced by an obesogenic diet, potentially linked to increased AF leptin levels. Further research is needed to explore the sex-specific effects.

## Introduction

The hormone leptin (16 kDa) is well known for its role in centrally regulating body weight and feeding behaviour, although its pleiotropic actions extend to the peripheral modulation of multiple biological processes, including the immune system, stress responses, bone growth, and vascular and reproductive functions^1,2^. Moreover, leptin also exerts crucial functions during foetal development, such as the regulation of embryo implantation, placental survival and proliferation, foetal angiogenesis and erythropoiesis, or the transport of nutrients across the fetoplacental unit^3–5^. Consequently, dysregulation of leptin production and/or function during foetal development may lead to altered growth^2^. In fact, in a rodent model, a high-fat diet before and during gestation has been associated with maternal adiposity, increased plasma leptin levels, and a 43% increase in foetal growth compared to their controls^6^. However, other studies have shown inconsistent results regarding maternal overnutrition and foetal growth^7^.

In humans, pregnancy is accompanied by a physiological increase in maternal circulating leptin levels, partly due to leptin produced and secreted by the placenta, and associated with a temporal maternal central leptin resistance, which seems to disappear immediately after parturition^2,8,9^. The rise of maternal leptin levels can be interpreted as an adaptation of the mother’s metabolism to meet the high energy demand of the developing foetus^10^. Despite the functional importance of leptin for foetal growth, an abnormal increase in leptin can trigger unfavourable pregnancy outcomes^11,12^. This is relevant because pregnant women with overweight or obesity have been documented to display significantly higher circulating leptin levels than normal-weight pregnant women^13^. In addition, it should be highlighted that amniotic fluid (AF) might be a direct source of leptin for the foetus at late gestation. In a rat model, we have previously described that leptin suddenly appears in AF at day 20 of gestation (since no presence of leptin was detected in the two previous days of pregnancy) and hypothesised that the AF leptin might have functional importance in the near-term foetus since it could be internalised into the immature stomach after AF swallowing^14^. Nevertheless, the potential developmental relevance of AF leptin and the consequences of alterations in its levels are still understudied.

Choline is an essential nutrient for foetal development with diverse pivotal functions^15^. This compound is a precursor (especially in the liver) of the universal methyl donor S-adenosylmethionine (SAM), which transfers one-carbon units for multiple physiological processes, including DNA and histone methylation reactions, with epigenetic impact^15,16^. Therefore, changes in perinatal choline handling could produce long-lasting effects on offspring by impacting epigenetic mechanisms. Moreover, animal studies have revealed that prenatal choline availability is crucial for optimal placental growth, nutrient transport, and macronutrient metabolism^17,18^. Increasing literature evidences the role of prenatal choline in the reversion or amelioration of maternal obesity malprogramming, as well as the normalisation of offspring metabolic phenotype^19,20^.

In the present study, we aimed to assess whether diet-induced maternal overweight/obesity, which is known to be associated with alterations in offspring metabolic programming^21–24^, causes changes in the production of leptin in the placenta and affects its levels in the AF, foetal gastric content and stomach, in both males and females, as well as its potential relation with foetal growth. The study has been performed at the last days of gestation (days 20 and 21), when leptin appears in the AF in our rat model, according to our previous study^14^. In addition, we also aimed to investigate whether maternal choline supplementation could counteract or ameliorate the potential adverse effects of maternal obesity. This study could provide new insights into the impact of maternal diet and key nutrients for development, such as choline, on the regulation of extraembryonic leptin levels, the possible amelioration of detrimental metabolic programming, as well as on sex-dependent differences in metabolic programming from early life.

## Results

### Body weight-related and food intake parameters of dams prior to mating and during pregnancy, and foetal body weight

During the four weeks of dietary intervention prior to mating, dams fed with western diet (WD) displayed higher cumulative energy intake (CEI), which was consistent with their higher fat mass and fat mass/fat-free mass ratio, and the lower free fat mass observed in this group, even though they showed no significant difference in body weight (Table 1). The CEI between days 1–19 of pregnancy was only significantly different between the groups at the p < 0.1 level (p = 0.072). The changes in body composition but not in body weight are consistent with increased adiposity in the WD-fed dams.

**Table 1:** Biometric and food intake parameters of dams.

	Standard chow diet	Western diet
Weight-related measurements prior mating		
Body weight (g)	221 ± 5	234 ± 6
Fat mass (%)	10.5 ± 0.9	16.8 ± 1.2*
Fat-free mass (%)	88.2 ± 1.0	82.8 ± 1.1*
Fat mass/fat-free mass ratio	0.12 ± 0.01	0.20 ± 0.02*
Cumulative energy intake (kcal)		
Before pregnancy (for 4 weeks)	1635 ± 26	2037 ± 43*
During pregnancy (days 0 to 19)	1291 ± 44	1403 ± 33

Body weight (g), fat mass (%), fat-free mass (%), and fat mass/fat-free mass ratio of 3-month-old female rats fed with a standard chow diet (CD; n = 5, a representative sample size of the group), and high-fat high sucrose (western) diet (WD; n = 8), prior mating. Cumulative energy intake of CD and WD-fed rats: during premating (accumulated from WD start until mating; 4 weeks) and during pregnancy (from day 0 to day 19 of pregnancy). *U*-Mann Whitney test was used for single comparisons between groups, and significant differences are indicated by *p < 0.05.

**Figure 1:**
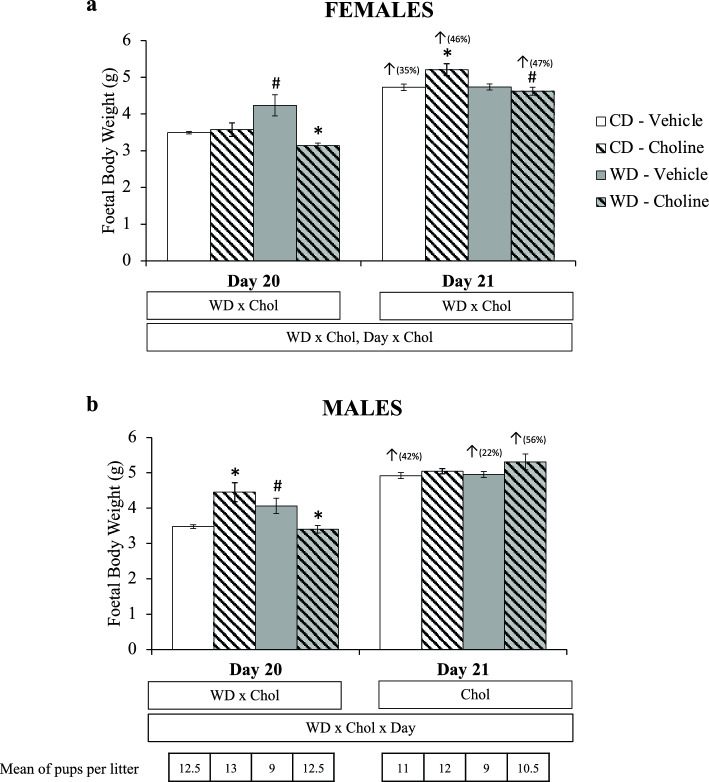
Foetal body weight (g) at gestational days 20 and 21, depending on maternal diet (standard chow (control) diet—CD—or western diet—WD) and maternal supplementation (vehicle or choline) of female (**a**) and male (**b**) foetuses. Data are mean ± SEM. Statistics: three-way (bottom box) and two-way (upper boxes) ANOVA with diet (WD), supplementation (Chol), and Day as factors. *U*-Mann Whitney test was used for single comparisons, with significant differences (p < 0.05) indicated by * (choline vs. vehicle), ^#^ (western vs. control diet), and ^↑^ (day 21 vs. 20; with the % of change between both days). Mean size of litters is given at the bottom part of the figure.

The weight of the foetuses depending on the day of gestation (20 or 21), maternal diet, and maternal choline treatment is shown in Fig. 1. It is worth mentioning that the weight of the foetuses can be affected by the size of the litter. Therefore, the mean number of foetuses in the litters of each experimental group is indicated. Interactive effects were observed by three-way ANOVA: in females, diet × treatment (WD × Chol) and day × treatment (Day × Chol); in males, diet × treatment × day (WD × Chol × Day). Analysing each day per separate, on gestational day 20, an interactive WD × Chol effect was observed by two-way ANOVA in both sexes. Both in females and males, maternal WD caused a significant increase in foetal weight, prevented by choline supplementation (Mann–Whitney *U* test). However, choline supplementation also caused increased body weight of male foetuses under maternal control diet. On day 21, diet × treatment interaction (in females) and the effect of treatment (in males) were observed (two-way ANOVA). In this case, maternal choline supplementation, under a control diet, caused increased weight of the female foetuses, an effect not observed in the WD animals. In fact, WD-choline females showed lower body weight than WD-vehicle females. In the case of males, a general effect of maternal choline treatment increasing foetal weight was observed (two-way ANOVA). Therefore, at day 21, the most remarkable effect caused by maternal conditions observed is the increase of foetal body weight by choline supplementation, except in the female offspring of WD-fed dams. The comparison of the foetuses’ body weight between days 20 and 21 also showed significant differences (Mann–Whitney *U* test) depending on the experimental groups and sex. In females, the weight was significantly increased in all groups except in the WD-vehicle group. In males, increased body weight was also observed in all groups except, in this case, for the control diet control diet (CD)-choline group. Interestingly, the groups where no significant weight increase was observed when comparing day 21 to day 20 foetuses correspond to those where the highest weight was reached at day 20 (i.e., WD-vehicle in females and CD-choline in males).

### Amniotic fluid, gastric content, and stomach leptin levels

**Figure 2:**
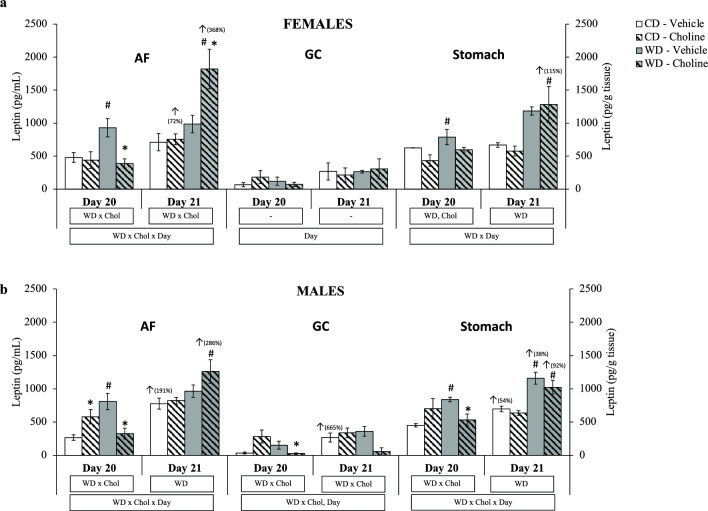
Leptin levels in amniotic fluid (AF), gastric content (GC) (pg/mL), and stomach (pg/g tissue) at gestational days 20 and 21, depending on maternal diet (standard chow (control) diet—CD—or western diet—WD) and maternal supplementation (vehicle or choline) of female (**a**) and male (**b**) foetuses. Data are mean ± SEM. Statistics: three-way (bottom box) and two-way (upper boxes) ANOVA with diet (WD), supplementation (Chol), and Day as factors. *U*-Mann Whitney test was used for single comparisons, with significant differences (p < 0.05) indicated by * (choline vs. vehicle), ^#^ (western vs. control diet), and ^↑^ (day 21 vs. 20; with the % of change between both days).

By three-way ANOVA, an interactive effect of WD × Chol × Day on AF leptin levels was found in both sexes. Regarding gastric content leptin levels, an effect of the day of gestation was found in females and males (basically, higher levels at day 21). However, an interactive effect of WD × Chol was also observed in males. In stomach leptin levels, interactive effects WD × Day and WD × Chol × Day were found in females and males, respectively (Fig. 2a,b).

At day 20 of gestation, the two-way ANOVA analysis showed a WD × Chol interactive effect in AF leptin levels in both sexes, and maternal WD (per se) was significantly associated with increased leptin levels in AF and foetal stomach in both sexes (Mann–Whitney *U* test). However, this increase was significantly reverted or prevented by maternal choline supplementation (except in the stomach of females, where the difference was only significant at the p < 0.1 level). The change was also reflected in the gastric content of WD males, where there was also a significant decrease in leptin levels by maternal choline treatment. Nevertheless, it must be noted that, in males, higher AF levels of leptin were found in the CD-choline in comparison to the CD-vehicle group.

At gestational day 21, the results of leptin levels in the AF show a WD effect in males and an interactive WD × Chol effect in females (two-way ANOVA). Essentially, the AF of female WD-choline foetuses showed substantially increased levels of leptin with respect to both WD-vehicle and CD-choline groups (Mann–Whitney *U* test), while in males, leptin levels in the AF were only significantly increased by maternal WD in the choline-supplemented group (Fig. 2a,b). Regarding the stomach, leptin levels at day 21 were significantly increased basically by maternal WD treatment in females and males (two-way ANOVA).

Considering the two days and treatments, it is worth mentioning that the general profile of changes in leptin levels was similar when comparing AF and stomach. Another remarkable fact comes when we focus on the increase of leptin levels from day 20 to day 21 of gestation (considering that different foetuses were analysed), with a different behaviour comparing females and males. In females, leptin levels were significantly increased from day 20 to day 21 in both AF and stomach in the WD-choline group (Mann–Whitney *U* test). However, in males, leptin levels increased significantly at day 21 in basal conditions (CD-vehicle group) in the three compartments (AF, gastric content, and stomach). When comparing day 21 to day 20, this increase in leptin levels was also observed in AF and stomach of the WD-choline males but not in gastric content. Also, a significant increase in stomach leptin levels was observed in the WD-control group on day 21 with respect to day 20.

Concerning leptin levels in gastric content, no significant changes were observed in female foetuses (except a Day effect in the three-way ANOVA), but males (of 20 days of gestation) followed a pattern of changes similar to that found in AF and stomach regarding maternal diet and/or choline supplementation, as explained above (Fig. 2a,b). In fact, at day 20, choline treatment caused lower leptin levels in the WD-group (Mann–Whitney *U* test). The increase of leptin levels between gestation days 20 and 21 was only significant in the CD-vehicle group but not in the WD-choline group (Mann–Whitney *U* test). Interestingly, and in accordance with previous results^14^, the gastric content levels of leptin were several-fold lower than in the AF, as well as compared to the stomach levels, in all the experimental groups, in agreement with the hypothesis that leptin from the swallowed AF might be internalised to the stomach.

### Leptin mRNA and protein levels in placenta

**Figure 3:**
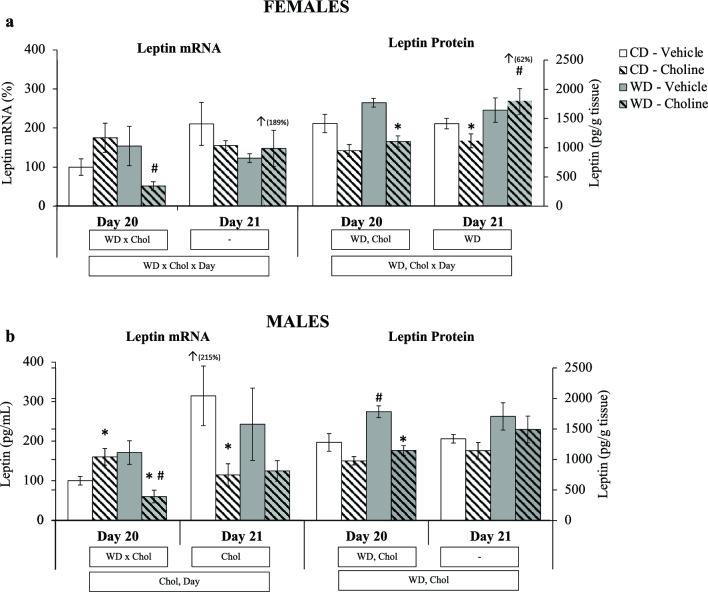
Placental leptin mRNA (% vs. control) and protein (pg/g tissue) at gestational days 20 and 21, depending on maternal diet (standard chow (control) diet—CD—or western diet—WD) and maternal supplementation (vehicle or choline) of female (**a**) and male (**b**) foetuses. Data are mean ± SEM. Statistics: three-way (bottom box) and two-way (upper boxes) ANOVA with diet (WD), supplementation (Chol), and Day as factors. *U*-Mann Whitney test was used for single comparisons, with significant differences (p < 0.05) indicated by * (choline vs. vehicle), ^#^ (western vs. control diet), and ^↑^ (day 21 vs. 20 with the % of change between both days).

Placental leptin gene expression was also analysed at the mRNA and protein level at days 20 and 21 of gestation (Fig. 3a,b). At day 20, the expression pattern was similar between males and females, showing a decrease of leptin mRNA expression in the WD-choline group (Mann–Whitney *U* test), a general increase in protein levels associated with maternal WD, and a decrease in protein levels given by maternal choline treatment (given by the two-way ANOVA analysis). Generally, and considering both sexes, at day 20 of gestation, the results of leptin expression both at the mRNA and protein level in the placenta show an effect of choline downregulating them in the offspring of WD-fed dams, while maternal WD (per se) would trigger a global effect of increasing leptin protein levels.

A different response to WD and choline supplementation was found on day 21. Essentially, the results show that maternal WD triggered an increase of protein leptin levels (but not at the mRNA level) in females (two-way ANOVA), which was not downregulated by maternal choline supplementation. However, there was a decrease of protein levels by maternal choline treatment in the CD group (Mann–Whitney *U* test). In males, maternal choline supplementation caused a decrease of placental leptin expression at the mRNA levels (two-way ANOVA), not reflected at the protein level. When comparing changes from day 20 to 21, it is remarkable the significant increase in leptin expression in females of the WD-choline group, both at the mRNA and protein level, as described above for leptin levels in AF and stomach (Fig. 2a), while in males, there was only an increase at the mRNA level in control (CD-vehicle) animals, as occurring for leptin levels in AF, gastric content, and stomach (Fig. 2b), but not observed in the other male groups.

These results show an expression pattern at the leptin mRNA level different from that of the protein. Furthermore, Pearson’s correlation analyses showed the absence of a significant correlation between placental leptin mRNA and protein levels (data not shown).

### Correlation of AF leptin levels with gastric, stomach and placental levels and with foetal weight

**Figure 4:**
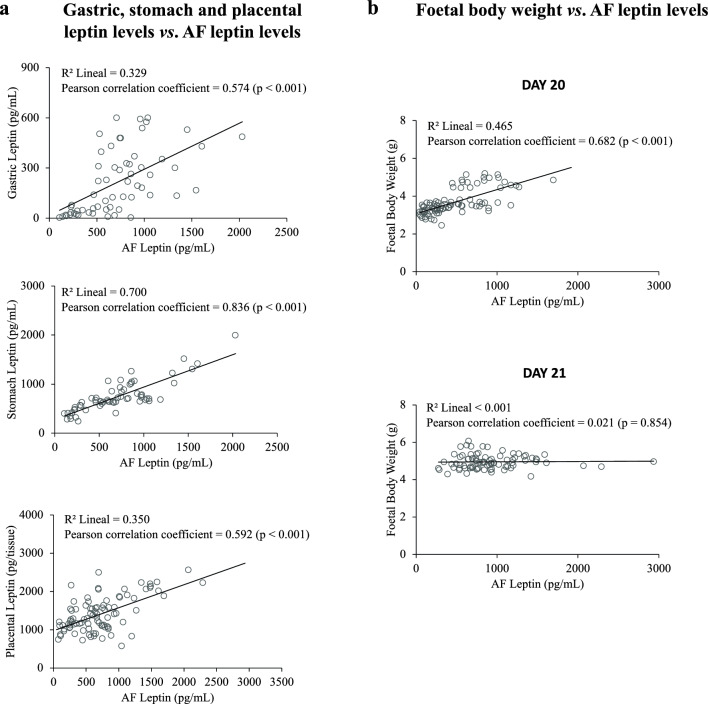
Correlation analysis between (**a**) gastric, stomach, and placental leptin levels and amniotic fluid (AF) leptin levels, and between (**b**) foetal body weight and AF at gestational days 20 and 21. In the case of gastric content and stomach, samples were pooled for leptin measurement (2–3 stomach samples per pool), and samples of the same pool shared the same value of leptin level for the correlation test. Statistics: lineal coefficient of determination (R^2^); Pearson correlation coefficient (and p-value) are given.

Figure 4a shows significant positive correlations, given by linear regression analysis, of AF leptin levels with gastric, stomach, and placental leptin levels. The highest correlation is given between AF and stomach leptin levels. These results are in accordance with previous results suggesting that leptin, mainly produced and secreted from the placenta, would pass into AF at late gestation and be internalised by the foetal stomach through AF swallowing by the foetuses^14^.

Foetal body weight was also positively correlated with AF leptin levels, considering all female and male foetuses in the analysis (when the analysis was done separating both sexes, the results were very similar—data not shown). However, the correlation pattern on day 20 is very different from that on day 21 (Fig. 4b). On the one hand, on day 20, the correlation between foetal body weight and AF leptin was positive and significant. In contrast, on day 21, the correlation between these two parameters was null, indicating a lack of dependence between the weight of the foetus and AF leptin levels at this day of gestation (just prior to parturition).

## Discussion

Maternal obesity has been associated with altered placental structure and function and foetal growth through alterations in maternal leptin production and signalling^2,25^. Moreover, extensive literature describes both short- and long-term detrimental effects on offspring of maternal obesity caused by overnutrition or consumption of the so-called westernised diet^21,23,24,26–28^. Our previous study^14^ gave new insights into the pleiotropic role of leptin during foetal development at late gestation since leptin was found to be present in AF (from day 20 in our model, i.e. near term) and a potential relationship between placental leptin production and leptin levels in AF, foetal gastric content, and foetal stomach was suggested, with AF leptin probably contributing directly to foetal stomach leptin^14^. We show here how maternal WD feeding, and/or the consequent increase in adiposity, affects the levels of leptin in embryonic and extraembryonic compartments near term, the potential consequences on foetal weight gain, and the possible reverting or protecting effects of maternal choline supplementation.

A positive correlation between the levels of leptin in AF and gastric content, stomach, and placenta near term was observed, supporting the relationship between leptin levels in AF and stomach during late pregnancy and the possibility that leptin coming from the placenta, through swallowed AF, can positively contribute to stomach leptin levels, as suggested before^14^. The presence of leptin in the stomach of near parturition foetuses and the observation of variations in its levels caused by specific gestational dietary interventions suggest a functional and local role of leptin in this very sensitive period of development.

In humans, it has been proposed that higher leptin levels around birth could act as a satiety signal until parturition, while, immediately after delivery, the sudden decrease of leptin levels might serve as a stimulus for feeding^29^. In adult rats and human subjects, stomach leptin released after food intake has been suggested to modulate satiety and feeding behaviour in the short term by targeting the CNS via peripheral nerves or the circulatory system^30–32^. Studies in lactating rats have demonstrated that leptin present in maternal milk can be absorbed by the immature stomach and transferred into the bloodstream, exerting its biological functions in target tissues^33^. Moreover, leptin has also been described as a neurotrophic factor able to regulate the formation of hypothalamic circuits^34^, which has been associated with the second week of life (in rats) when the leptin surge is given^34,35^. However, we cannot discard key leptin actions in other fundamental development windows. Stomach leptin obtained from the swallowed AF may be transferred to foetal circulation during late gestation and induce satiety or even modulate neuronal circuits via its action in the developing CNS, especially the hypothalamus, a main responsible for the control of food intake and energy expenditure throughout life^36^. Along these lines, environmental factors modulating such leptin levels might be crucial for later health-related outcomes and energy metabolism. In addition, we have hypothesised that leptin obtained from the AF might affect foetal growth, and excessive leptin supply may alter the normal growth pattern. Specifically, we show here that maternal WD increases leptin levels in AF and foetal stomach at gestational day 20, and the increase is prevented by maternal choline supplementation. Moreover, at day 20, WD-vehicle foetuses showed higher body weight (both males and females) than CD-vehicle group, but not WD-choline foetuses, and differences due to maternal WD were no longer observed on day 21. Therefore, choline supplementation could be causing a preventive effect of the increased foetal weight gain caused by maternal WD feeding. Considering the positive correlation between AF leptin levels and foetal weight at day 20 found here, we suggest that the increased leptin levels in the placenta and AF associated with maternal WD feeding might be related to the increased foetal growth at this day of gestation. As previously suggested^14^ leptin discharged by the placenta to the AF, could be taken by the foetal stomach by AF swallowing and followed by subsequent internalisation. Likewise, such variations in leptin levels in AF, and consequently in the stomach, might putatively modify the physiological satiety capacity or affect hypothalamic development of the near parturition foetus, a suggestion that deserves further research. The normalisation (partly or entirely) of the maternal WD-increased levels of leptin in AF and stomach by maternal choline supplementation might be interpreted as a reversion or prevention, at least in part, of the maternal WD effects on leptin levels in these compartments, at day 20 of gestation. It has to be noted that, under a maternal control diet, choline supplementation per se is also associated here with increased foetal body weight at day 20 in males and with increased AF leptin levels. Moreover, maternal choline supplementation led to a higher increase in body weight among female foetuses between days 20 and 21, provided that the foetuses had not experienced premature growth by day 20. This effect resulted in a greater weight at day 21 for females on a control diet compared to those whose mothers had not received supplementation. The reasons why choline has different effects depending on the sex of the animals, particularly under a control diet, as well as the significance of these effects and their potential impact on subsequent growth, deserve further investigation. It is worth mentioning that the body composition of the foetus has not been determined. In the case of rats, adipose tissue develops during lactation, and the amount of fat during the foetal stage is practically negligible^37^. However, we cannot rule out a possible effect of choline on increasing foetal adiposity. The pattern of leptin in foetal and extraembryonic compartments at day 21 of gestation (just prior to parturition) is somewhat different, and changes in leptin levels may be of interest since this is a critical moment of preparation for parturition. On this day, although there was an overall effect of maternal WD increasing stomach leptin levels, AF leptin levels were found to be increased, particularly in the WD-choline group. This sudden increase from day 20 to 21 (368% and 286% in females and males, respectively) could be related to the significant difference in body weight when comparing the 21-gestational day foetuses to the 20-gestational day ones (47% and 56% in females and males, respectively). AF leptin levels also increased significantly from day 20 to 21 in the female foetuses of the CD group supplemented with choline (72%) and in the male foetuses of the CD group treated with vehicle (191%). Both groups of foetuses also experienced a significant increase in their weights (46% and 42%, respectively), with CD-choline females reaching values significantly higher than those of the control group. However, unlike on day 20, no direct correlation was found between AF leptin levels and foetal weight on day 21, considering all experimental groups. This lack of correlation probably reflects the preponderance of other regulatory factors at times closer to delivery. This might also be related to the pleiotropic role of leptin during late gestational foetal development. Besides influencing foetal growth and development, an effect particularly observed on day 20, its satiating function could be more crucial on day 21, just before and during parturition. In general, this study provides new evidence that supplemental choline given to dams from day 11 to 17 of gestation would impact different foetal and extraembryonic compartments, modulating the changes caused by a harmful condition, such as maternal intake of an obesogenic diet. Interestingly, the effects of maternal choline supplementation depend on the sex of foetuses. Regarding placental leptin production, the measured levels at day 20 in male and female foetuses indicate two general effects: a downregulation of protein leptin levels by maternal choline treatment and an upregulation by maternal WD feeding. In fact, the choline effects were enough to revert the increased leptin levels caused by maternal WD feeding. These changes might have an impact on the placenta functioning since the placenta is finely regulated by self-produced leptin^38^. Again, at day 21 the situation is different, as the increase in leptin levels by maternal WD diet in females, is not reverted by choline supplementation. Placental leptin protein seems to be regulated by other factors beyond the endogenous leptin mRNA expression on both gestational days, as the pattern of leptin protein levels differed from that of mRNA. This observation is further supported by the lack of correlation between placental leptin mRNA and protein levels (data not shown), suggesting that other factors might be modulating leptin protein levels found in the placenta, such as posttranscriptional regulation and the regulation of its transport from the placenta to the AF.

Overall, the results show that foetal (stomach) and extraembryonic (placenta and AF) leptin levels are susceptible to modulation by both maternal obesogenic diet and choline supplementation. This is seen more clearly on day 20 of pregnancy than on day 21, which is more influenced by the events of parturition. Here we have shown that maternal WD leads to higher leptin levels in the placenta, AF, and stomach of 20-day-old foetuses, and that maternal choline supplementation prevents most of these changes. Given that maternal WD and increased adiposity may be considered a detrimental condition, and previously linked to early poor metabolic programming^39,40^, choline supplementation limiting the changes caused by WD feeding during Pregnancy may be beneficial for the offspring. On the other hand, although the current results support the idea that AF leptin comes, at least in part, from the placenta and can affect foetal weight, the mechanism involved in this transport is not yet known and deserves further investigation. It is also intriguing whether the WD- or choline-dependent changes observed in leptin levels in late gestation may affect foetal programming of obesity. 

While this study has characterized the impact of choline supplementation and maternal obesogenic diet on leptin levels in the AF, it also paves the way for future research in this area. On the one hand, there are effects of maternal supplementation that are not feasible to be studied with the current experimental design and remain to be explored, including the potential modulation of leptin levels in the stomach and body weight of the offspring during the suckling period. Another aspect that might deserve future studies is the meaning of the pronounced increase of leptin levels in AF observed on day 21 in the offspring from dams supplemented with choline and fed a WD. On the other hand, the results of this study provide a basis for experimental designs to characterize the long-term effects on offspring of mothers supplemented with choline during pregnancy and the potential interaction with the maternal diet. Although most prenatal choline supplementation studies focus on cognitive benefits in offspring, both in rodents^41,42^ and in humans^43,44^, there is evidence to suggest that gestational choline supplementation is associated with improved long-term regulation of offspring biomarkers of the metabolic syndrome, especially in male rodents fed a high-fat diet^45,46^. Hence, further research is essential to characterize additional mechanisms underlying the long-term beneficial health outcomes associated with choline supplementation, such as the epigenetic modifications caused by prenatal exposure, and assess their long-term durability.

## Methods

### Animal experimental design

The animal protocol followed in this study was reviewed and approved by the Animal Experimentation Ethics Committee (CEEA) of the University of the Balearic Islands (resolution number CEEA 86-02-18), following the institutional use and care guidelines for laboratory animals. Seventeen two-month-old female Wistar rats (Envigo, Barcelona, Spain) were used and divided into standard chow diet (CD) (Chow diet A04, SAFE diets, Augy, France) or high-fat and high-sucrose western diet (WD) (D12079B, Research Diets, New Brunswick, USA) fed groups. After four weeks of dietary intervention, all rats underwent a body composition analysis by EchoMRI-700™ (Echo Medical Systems, Texas, USA) to ensure that, prior to mating, WD produced obesogenic diet-related changes in the corresponding rats. Food intake was recorded during the dietary intervention. Female rats were mated with male rats, and pregnant rats were housed apart. Conception day (day 0 of gestation) was confirmed by the detection of sperm in the vaginal smear. Maternal body weight was recorded until euthanasia to monitor pregnancy evolution. Both CD and WD pregnant rats were randomly distributed into water (vehicle) or choline-treated dams and into a specific day of euthanasia, day 20 or 21 of gestation. Eight experimental groups were produced after combining all the factors mentioned above, namely, the female and male offspring of CD-fed dams treated with vehicle (CD-vehicle) or supplemented with choline (CD-choline) and of WD-fed dams receiving vehicle (WD-vehicle) or choline (WD-choline). Dams were euthanized by CO_2_ asphyxia in the corresponding gestational day under ad libitum feeding conditions at the beginning of the light cycle. The foetuses on both days 20 and 21 were obtained by caesarean section, weighed and euthanized by decapitation, and AF, umbilical cord, placenta, foetal stomach, and gastric content of each foetus were collected, together with foetal liver (for DNA isolation), frozen immediately in liquid nitrogen and stored at −80 °C until analysis. The gastric content was collected by sucking the fluid inside the stomach with a BD Micro-Fine syringe coupled to a 30 G needle (Becton, Dickson and Company, New Jersey, USA) due to the small size of the stomach. The stomach of foetuses was rinsed with saline containing 0.1% diethylpyrocarbonate.

### Choline supplementation

For maternal choline supplementation, choline chloride (Sigma Aldrich) was dissolved in water (vehicle) and was given by oral gavage from day 11 to day 17 of gestation (a specific period in which choline bioavailability is essential for proper cognitive development^47^). The selection of the dose of choline was based on rodent prenatal choline treatment studies^47–50^. Thus, choline supplementation was four times the daily amount of choline ingested with the diet, calculated based on the intake of dams under CD (estimated from the average intake of the female rats on the day before mating). It is worth mentioning that the choline content per gram of diet is somewhat higher in the WD diet compared to the CD (2.0 mg/g vs. 1.6 mg/g). However, this difference is partly compensated by the fact that the amount of diet ingested by the WD animals is lower compared to that of the CD animals due to their greater satiating effect and caloric content. Therefore, 126 mg of choline chloride were dissolved in 3 mL of water (volume adjusted to choline chloride solubility, 50 mg/mL, and the maximum volume of administration allowed in rats according to the guidelines followed in this study). The same volume of water was administered in the same way to the non-supplemented group.

### Foetus sexing

Foetal sex was first characterised by anogenital distance and genetically confirmed by qPCR amplification of a particular region of the sex-determining region Y (*Sry*) gene in liver samples. For liver DNA extraction, around 20 mg of foetal liver were added to 75 µL of an alkaline solution containing 25 mM NaOH and 0.2 mM EDTA, and the mix was heated at 98 °C for 1 h in the 2720 Thermal Cycler (Applied Biosystems, Massachusetts, USA) to disaggregate the tissue and release the DNA. Cellular debris was removed by centrifuging the homogenate at 4000 rpm for 3 min at room temperature after heating. DNA-containing supernatant was collected and purified by ethanol precipitation. The DNA sample was mixed with cold absolute ethanol 1:2 (v/v) and centrifuged at 16,000*g* for 15 min at room temperature. The supernatant was discarded, and the resulting DNA pellet was washed with 1 mL of 70% ethanol. Finally, ethanol was removed, and the DNA was re-suspended in 100 µL of RNase-free. Besides *Sry*, the β-actin gene (*Actb*) was also amplified to decrease the cases that actual male samples are not mistakenly identified as female when *Sry* gene amplification was not produced. In other words, it is a method to ensure that the lack of *Sry* gene amplification is not due to unsuccessful DNA extraction or PCR performance but only caused by sex condition.

### Leptin quantification

Leptin levels were quantified in the placenta, AF, gastric content and foetal stomach with a commercial Mouse/Rat Leptin Quantikine ELISA kit (R&D Systems, Minnesota, USA). The details of the sample processing for ELISA assaying are described elsewhere^14^.

### RNA isolation and quantitative reverse transcription polymerase chain reaction (RT-qPCR) analyses

Total RNA was isolated from the placenta using the commercial EZNA^®^ TOTAL RNA kit I (Omega Bio-Tek, Georgia, USA), as the manufacturer’s protocol describes. For that purpose, 10–20 mg of placenta were homogenised in 700 µL of TRK Lysis Buffer using a Polytron homogeniser under cold conditions to avoid sample degradation during homogenisation. Then, 250 ng of total RNA were also used for RT and denatured at 65 °C for 10 min in the 2720 Thermal Cycler. In this RT procedure, 7.5 µL of the RT-mix containing: 2.5 µL of 5× First Strand Buffer, 2 µL of dNTPs as above, 0.5 µL of random hexamers, 0.5 µL of RNase Inhibitor, 0.5 µL of enzyme Reverse Transcriptase, and 1.5 µL of RNase free water, were added to the denatured RNA. The RT was conducted in the 2720 Thermal Cycler (Applied Biosystems, Massachusetts, USA) with the following conditions: 10 min at 25 °C, 50 min at 37 °C and 15 min at 70 °C. Thus, cDNA was obtained to measure mRNA expression levels of *Lep,* and *β-actin* was used as the reference gene. Designed primers for *Lep* (forward: aggaaaatgtgctggagacc; reverse: ataccgactgcgtgtgtgaa) and *Actb* (forward: tacagcttcaccaccacagc; reverse: tctccagggaggaagaggat) were purchased from Sigma Genosys (Sigma Aldrich Química SA, Madrid, Spain). For each PCR, 2 µL of the diluted cDNA and 9 µL of PCR mix containing 5 µM of each forward and reverse primers and ready-to-use Power SYBER Green PCR Master Mix (Applied Biosystems, Massachusetts, USA) were used under the following protocol: 10 min at 95 °C, followed by 40 two-temperature cycles (15 s at 95 °C and 1 min at 60 °C). A product-specific melting curve was obtained to verify the purity of the PCR products after each run. The cycle of threshold (Ct) was calculated by the StepOnePlus™ Software v2.2.2 (Applied Biosystems, Massachusetts, USA), and the relative mRNA expression was calculated as a percentage, where 100% of mRNA expression of each gene was set both to control male and female offspring, using the 2^−ΔΔCt^ method^51^.

### Statistical analysis

All data are reported as mean ± standard error of the mean (SEM). Stomach leptin values were weighted in the statistical analysis by the number of samples pooled during the experimental analysis. For multiple comparisons, three-way analysis of variance (ANOVA) considering gestational day (day 20 and day 21), diet (CD and WD), and treatment (vehicle and choline), and two-way ANOVA considering diet and treatment were performed. The Shapiro–Wilk test was used to test the normality of the data distribution. Because of the non-normal nature of the data distribution, and the samples’ size (n < 10 in some cases), the *U*-Mann Whitney test was used for single comparisons. The Pearson Correlation test was used to assess the linear dependence between two variables. The statistical test and symbols used for each comparison are described in the footnotes of the figures and tables. The significance threshold was set at p < 0.05. Analyses were performed with SPSS for Windows (SPSS version 27.0.0, Illinois, USA).

### Ethics approval

The animal experimental protocol was approved by Animal Experimentation Ethics Committee of the University of the Balearic Islands (resolution number CEEA 86-02-18). The protocol of this study is reported in accordance with ARRIVE basic guidelines. The dams of this study were obtained from Envigo (Barcelona, Spain).

## Data Availability

Data is available from the corresponding author on reasonable request.
